# Annotating patient clinical records with syntactic chunks and named entities: the Harvey Corpus

**DOI:** 10.1007/s10579-015-9330-7

**Published:** 2016-01-11

**Authors:** Aleksandar Savkov, John Carroll, Rob Koeling, Jackie Cassell

**Affiliations:** 1 Department of Informatics, University of Sussex, Brighton, BN1 9QJ UK; 2 Division of Primary Care and Public Health, Brighton and Sussex Medical School, Brighton, BN1 9PH UK

**Keywords:** Corpus annotation, Annotation guidelines, Clinical text, Chunking, Named entities

## Abstract

The free text notes typed by physicians during patient consultations contain valuable information for the study of disease and treatment. These notes are difficult to process by existing natural language analysis tools since they are highly telegraphic (omitting many words), and contain many spelling mistakes, inconsistencies in punctuation, and non-standard word order. To support information extraction and classification tasks over such text, we describe a de-identified corpus of free text notes, a shallow syntactic and named entity annotation scheme for this kind of text, and an approach to training domain specialists with no linguistic background to annotate the text. Finally, we present a statistical chunking system for such clinical text with a stable learning rate and good accuracy, indicating that the manual annotation is consistent and that the annotation scheme is tractable for machine learning.

## Introduction

Clinical text in primary care electronic patient records is a source of rich, detailed information that could be of great use for health service planning and for the study of disease. However, unlocking that information at scale for research purposes is hindered by processing difficulties caused by the peculiarities of clinical language use, and a lack of development data due to the presence of sensitive information. The main short term goal of most research in the area is to achieve a reliable language processing foundation to allow more complex tasks such as named entity recognition (NER) to reach a sufficiently reliable performance level. Achieving this goal would allow recognised semantic entities to be associated with presence, absence, or degree of certainty, and other attributes such as history of a health condition, etc. If such processing tasks reach a certain level of reliability, they could be used to avoid manual information extraction from clinical text and the manual de-identification that is currently required.

**Fig. 1:**
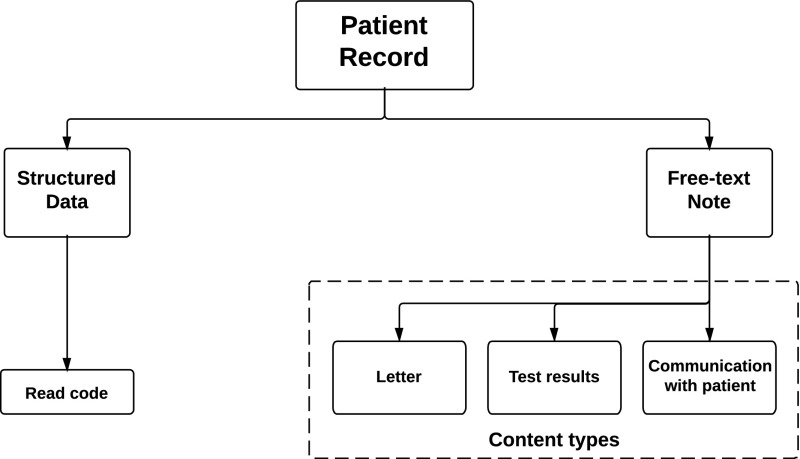
Patient record content diagram

Most general practices in the UK use software packages to store and maintain their electronic health records (EHR) in structured form. The data is collected in several databases, the General Practice Research Database (GPRD)^1^ is one of the largest, with high quality data and linkages to other UK datasets, hosted in the UK’s Medicines and Healthcare Regulatory Agency. While great efforts are being made to process, interlink, and reuse the structured part of primary care patient records (see Fig. 1) with other secondary care data, very little has been done to exploit the information in the free text notes. The details about symptom and disease typed in by the general practitioners (GP),^2^ have not only the potential to enrich their structured counterparts, but in many cases they can be the only source of relevant information. The latter is well illustrated by structured (coded) data entries such as *Had a chat to patient* and *Telephone encounter*, which carry no medical meaning, and rely solely on the information recorded in the text of the examination record to convey details about the patient encounter (see Table 1).

**Table 1:** Examples of examination records from the GPRD consisting of a structured entry (*left*) and a text note (*right*)

Telephone encounter	tel from wife pt v scared re mri next wed- ok for small dose dz
Constipation NOS	1 BM 3 days ago following 5 days without any. now no BM last 3 days either. breast fed baby ! o/e abd soft. no palpable faeces. try lactulose 2.5 ml bd
Cardiac failure therapy	Hxnsyx settled ? feels abit better OE creps R base only. jvp not seen. IMP better re fluid status, rate still ok. P cont w bloods 2/7, rv 1w
Had a chat to patient	re. cough at night; see letter from Mr ~~~~~

A few studies have successfully made use of UK primary care clinical notes primarily using heuristics and rule-based algorithms taking advantage of regularities of the data in particular sub-domains (Koeling et al. 2011; Shah et al. 2012). However, such methods are difficult to transfer even to a slightly different type of data or task. To avoid these kinds of obstacles, current research in Natural Language Processing (NLP) focuses on more generic Machine Learning (ML) methods. To date no significant advances have been made in employing such methods on primary care clinical text, mainly because of its non-canonical language, which is different from the edited text normally considered in mainstream NLP research. The language is characterised by extreme brevity of thought and expression, numerous medical terms and jargon, ungrammatical constructions, spelling mistakes, and irregular and unorthodox usage of punctuation. These qualities of the data require a test set with gold standard annotation (ground truth) if the off-the-shelf NLP tools are to be evaluated fairly. Moreover, successful use of any machine learning-based method requires an adequate quantity of annotated clinical text for training purposes. Certain similarities exist between primary care text notes and some types of secondary care data such as radiology reports. However, using tools trained on such data would be at a significant cost mainly due to the greater variety of topics and vocabulary used in general practices as a whole, compared to radiology reports for instance.

Ideally gold standard datasets should be developed cumulatively in the order of core NLP processes—tokenisation, word normalisation, sentence segmentation, Part of Speech (POS) tagging, syntactic parsing. However, considering the challenging qualities of the data and the limited time and funding at our disposal, a successful first step had to aim to create a gold standard that contribute the most, regardless of its place in the order. The output of various state-of-the-art tools specialising in each task was analysed in order to make a decision. Even though their performance was not measurable without a gold standard it could be seen it was below the accuracy figures usually reported. However, due to their complexity the efforts needed to develop the first three tasks significantly outweighed the potential benefits of their improvement. We established that state-of-the-art POS-taggers perform to a reasonable level, reaching 0.82 F_1_-score evaluated on a small subset of our data (Savkov et al. 2014). However, we suspected that due to the sparsity of the data it would be difficult to improve their performance munch beyond this, especially given the significant proportion of tokens occurring once or twice in the whole corpus. Additionally, the chunking output showed more robustness and potential for improvement than dependency and constituent parsing, which are heavily affected by missing words and lack of sentence boundaries. Therefore creating a chunking gold standard was considered the most beneficial option.

We also considered adapting the methods for dealing with erroneous sentences suggested by Foster (2007) in order to develop full syntactic annotation, but we decided against it, because even if erroneous sentences are handled, sentence boundary identification remains a problem. A method implemented by Fan et al. (2013) follows this approach accounting for missing and spurious words by adding special nodes in the annotation. It also simplifies the internal structure of some phrases, making them flatter to avoid errors caused by ungrammatical expressions. We see this approach as only slightly more complex than chunking, but still probably less suitable for our data. Our analysis concluded that chunking identifies enough syntactic structure to support NER, while it can be achieved through sequence labelling, which reduces the negative effects of missing words and sentence boundaries.

Here we present the Harvey corpus, a de-identified corpus of clinical text annotated with syntactic chunks and semantic entities.^3^ Following previous research in annotation of clinical text (Roberts et al. 2008; Fan et al. 2011), we developed a set of annotation guidelines and trained four domain specialists to use them on primary care text. Two specialists annotated the corpus data independently, after which the third merged their annotations following a specific set of rules for resolving annotation conflicts without adding new information. We evaluate this annotation extrinsically by training a statistical chunking model and a semantic entity recognition model using a widely used statistical tagger.

This article is organised as follows. Section 2 describes other corpora in the biomedical domain and related research working with clinical notes. Section 3 gives a detailed description of the GPRD and the specific qualities of primary care notes. Section 4 describes the design and development of the corpus annotation, including the scheme, guidelines, and the training of annotators. Section 5 outlines the process of building the Harvey corpus, the data selection, the assembly of annotation layers, and the final resource. Section 6 evaluates the corpus extrinsically by using it as training and testing data in two practical tasks. Finally, Sect. 7 summarises the work and considers possible future directions.

## Related work

In this section we summarise two areas of research relevant to this study. Firstly, we present a list of corpora resources in and close to the clinical domain (Sect. 2.1), and secondly, we present recent research giving concrete examples of the knowledge contained in the GPRD data and illustrating the potential that could be unlocked by better NLP tools (Sect. 2.2).

### Related corpora

The term *biomedical corpora* is generally used to refer to text data collections from the life sciences. They typically cover a very wide range of studies and types of annotations, but generally keep to sources of scientific writing commonly found through MEDLINE and PubMed. Processing such corpora using tools trained on generic news text could be difficult due to the specific scientific, terminology-rich language of their origin. Therefore they also focus on linguistic annotation that allows the testing and development of core NLP tools better suited to that domain. Given the similarities discussed above, we think it is important to review here some corpora with more widely recognised impact in the field. In addition, Verspoor et al. (2012) provide a link to a more comprehensive list of publicly available corpora in the domain.^4^

GENIA (Ohta et al. 2002) and GENETAG (Tanabe et al. 2005) are two of the best established and widely applied biomedical resources. They both contain protein and gene annotation, providing a solid base for Information Extraction (IE) research. GENIA was manually annotated by domain experts using an ontology developed in parallel with the annotation process. Tanabe et al. (2005) describe GENETAG, which was created using an automated approach to ensure a balance between negative and positive examples. They used a Naïve Bayes classifier to determine the likelihood of a document containing a gene or protein name. The selected sentences were tagged with the AbGene tagger (Tanabe and Wilbur 2002) and finally the annotations were manually transformed by three domain experts. The Colorado Richly Annotated Full-Text (CRAFT) corpus (Cohen et al. 2010) is a more recent resource that contains 97 Open Access journal articles with syntactic, coreference, and concept annotations. Initially coreference was annotated using a modified version of the OntoNotes guidelines (Hovy et al. 2006), but later Verspoor et al. (2012) added syntactic annotation following the Penn Treebank guidelines (Bies et al. 1995) and the BioIE addendum (Warner et al. 2004). At the same time, concept annotation was added, identifying all mentions of nearly all concepts from nine prominent biomedical ontologies and terminologies (Bada et al. 2012).

During the past ten years a number of *clinical corpora* have been developed by the NLP community, thereby facilitating many of studies in the area (see Table 2 for a non-exhaustive list), and although data access is still a considerable problem, shared tasks and challenges have played an important role in the development of the field, providing relatively easy access to the same resources to a wider range of scientists. Perhaps the most notable such group is the i2b2 series, which also included a community annotation task. Uzuner et al. (2010b) present a set of guidelines for the annotation of seven attributes associated with medications in discharge summaries. The guidelines were developed through an iterative process during which a group of medical students annotated a few discharge summaries and provided feedback used for revision. The guidelines were used in the i2b2 community annotation experiment, comparing the inter-annotator agreement (IAA) (measured in F_1_-score) of community annotator teams and expert annotator teams. The authors found that the IAA of the two teams is comparable, and concluded that involving the community in fairly complex annotation processes is an acceptable alternative to using domain experts. The second part of the task was to automatically extract medication information (Uzuner et al. 2010a). The rest of the i2b2 challenge corpora were provided to the community in order to promote research in particular areas. Uzuner et al. (2007b) show the results of the participating automatic de-identification systems, trained and evaluated on a corpus of 889 de-identified discharge summaries. A subset of that corpus containing 502 summaries was also annotated with patient smoking status for the purposes of one of the challenge subtasks (Uzuner et al. 2007a). Another i2b2 challenge was aimed at identifying obesity and its comorbidities in clinical text using a corpus of 1237 discharge summaries (Uzuner 2009). A subset of this corpus was later annotated with entities and relations pertinent to congestive heart failure as part of the PhenoCHF corpus (Alnazzawi et al. 2014). The 2010 i2b2 challenge focused on identifying medical concepts, assertions, and relations (Uzuner et al. 2011). The organisers provided the participants with 871 discharge summaries annotated accordingly. Finally, a corpus of 310 discharge summaries annotated with temporal relations were provided for the latest i2b2 challenge (Sun et al. 2013). The data annotation of all challenge corpora kept to using two independent annotators and an adjudicator when possible. However, it is interesting to note that the adjudicators of the last challenge corpus were also allowed to edit or remove annotations in cases where the other annotators disagreed.

**Table 2:** A non-exhaustive list of notable clinical corpora

Corpus	Size	Document type	Annotation type
Harvey Corpus	750	GP notes	Syntactic chunks, four semantic annotation types
Uzuner et al. (2007b)	889	Discharge summaries	De-identification, smoker status
Uzuner (2009)	1237	Discharge summaries	Present, absent, questionable for obesity + 15 comorbidities
Uzuner et al. (2010b)	1243	Discharge summaries	Medications, dosages, frequencies, modes, reasons, durations, list/narrative
Uzuner et al. (2011)	871	Discharge summaries, progress reports	Concepts, assertions, relations
Sun et al. (2013)	310	Discharge summaries	Temporal relations
Roberts et al. (2009)	565 k	Histopathology reports, clinical narratives, and imaging reports	Entities and relations
Pakhomov et al. (2004)	271	Clinical notes	POS
Ogren et al. (2008)	160	Outpatient notes	Concepts from a subset of SNOMED-CT
Voorhees and Hersh (2012)	17 k	Patient visits consisting of history and physical reports, surgical pathology reports, radiology reports	Topics
Pestian et al. (2007)	1954	Radiology reports	ICD-9-CM codes
Fan et al. (2011)	50	Progress reports	POS
Fan et al. (2013)	25	Progress reports	Syntactic trees of ill-formed sentences

Note that the size is reported in terms of number of documents

Other shared tasks have focused on document level annotation of clinical corpora. The TREC 2011 and 2012 conferences Medical Records tracks used 17,264 clinical documents of various types from the University of Pittsburgh NLP repository for a topic modelling task (Voorhees and Hersh 2012). Pestian et al. (2007) present a small corpus of radiology reports annotated with ICD-9-CM codes.

The CLEF corpus (Roberts et al. 2008, 2009) is another prominent clinical text resource built to assist the development and evaluation of an IE system as part of a larger framework for the capture, integration and presentation of clinical information. The corpus includes 565,000 de-identified records of 20,234 deceased patients of the Royal Marsden Hospital oncology centre. An annotation scheme was developed using a cyclic process of annotating, analysing and improving. The records were first annotated by two medical domain experts and then the two sets of annotations were adjudicated by a third medical expert.

Few studies have focused on dealing with core NLP issues such as POS tagging and parsing of clinical text. Pakhomov et al. (2004) describe the annotation of 271 clinical notes (100,650 tokens across 7299 sentences) using the Penn Treebank guidelines Santorini (1990), achieving 87.95 % average agreement between three medically trained annotators calculated using Cohen’s *kappa* (Cohen 1960). More recently, Fan et al. (2011) presented two sets of 25 annotated progress notes from Kaiser Permanente Southern California and the University of Pittsburgh Medical Center, a subset of the i2b2/VA challenge. They were annotated with POS tags for the purpose of developing and evaluating POS tagging models. The corpus comprises 31,400 tokens in 3283 sentences annotated using a modified version of the original Penn TreeBank part-of-speech tagging guidelines (Santorini 1990). A subsequent study on part of the same data presented a set of guidelines for syntactic parsing of ill-formed clinical sentences, and a Treebank of 1100 syntactically annotated sentences from the i2b2/VA challenge (Fan et al. 2013). The presented guidelines are an extended version of the original Penn TreeBank II bracketing guidelines (Bies et al. 1995). They were modified to help the annotators handle the non-canonical language of clinical text by flattening certain syntactic constructions, introducing a mechanism for handling omitted words, and addressing other lesser issues in clinical text. The authors report IAA F_1_-score reaching 0.93 on the final set of 450 sentences and parsing performance F_1_-score reaching 0.81 using a statistical model trained on mixed data (newspaper and clinical text).

### Research using UK primary care data

The information in UK primary care records is an important medical research resource, but so far only a small fraction of its free text part has been extracted and used. Some of the first studies in this area show that the information in the free text has great potential (Shah et al. 2012; Koeling et al. 2011).

The Freetext Matching Algorithm (Shah et al. 2012) is an automated method for extracting information from free text. The algorithm uses dictionaries of Read code terms (Bentley et al. 1996) and “regular” words, as well as spelling correction software to make the language more canonical. Then it uses synonym look-up tables and phrase patterns to identify diagnoses, dates, and selected test results. The algorithm creates approximate matches between words and expressions in the free text on one side, and Read and OXMIS^5^ codes on the other. It was tested on two sets of 1000 records—one general and one associated with death—each taken from the GPRD. The algorithm achieved 0.98 precision and 0.93 recall on the death related dataset, and 0.92 precision and 0.77 recall on the other dataset. The authors also presented a cause of death detection algorithm aided by the Freetext Matching Algorithm to address the cases of cause of death recorded only in the free text. They conclude that the algorithm has achieved sufficient precision and it may facilitate research using patient record free text, particularly for extracting cause of death.

Koeling et al. (2011) annotated the records of 344 women in the year prior to an ovarian cancer diagnosis, and developed a method for automatic symptom detection in free text notes. The study was aimed at finding the incidence of five common symptoms of ovarian cancer. The estimated incidence of of each symptom in the manually tagged text was at least 40 % points higher than the structured data alone. The automatic method developed for the study was able to extract a significant proportion of this information (0.46 recall) with high precision (0.96). The automated approach was intended to aid medical researchers who wish to validate studies based on codes, or to accurately assess symptoms, using information automatically extracted from free text.

## GPRD data

We have created the Harvey corpus by annotating de-identified data from the General Practice Research Database, a database of longitudinal primary care medical records. The database contains comprehensive observational data from general practices, which makes it a valuable resource for a broad range of research areas, such as clinical epidemiology, disease patterns, disease management, research outcomes, and drug utilisation. Its data is gathered from primary care medical records where GPs and other health workers input information on events regarding their patients as structured data and free text. Structured data varies among the several software systems certified by the National Health Service (NHS), however, a Read code (Bentley et al. 1996) and a term associated with it are always present in each record (see Fig. 1). The Read codes are a clinical terminology system used in NHS primary care.^6^ The system goes beyond the expressive power of diagnosis encoding, being able to encode a wide range of patient phenomena, not specifically restricted to clinical terminology, such as administrative items, social circumstances, ethnicity, and religion.

The language and content of the free text is related to the role of GPs in the NHS. They are the gatekeepers to specialist care, charged with basic care for patients, and initial assessment and recommendation for specialist treatment. They are organised in small practices of several practitioners set up independently from the hospital system. Apart from the correspondence with specialists, GP notes are mainly intended for use within the same general practice they were created at.

The free text notes discussed in this study were obtained under a license with a programme of research *The Ergonomics of Electronic Patient Records* funded by the *Wellcome Trust*. They fall into three major categories: letters to and from specialists; test and scan results; and general notes of a patient visit or interaction (see Fig. 1). The letters are usually very descriptive and detailed, grammatically well written, and generally meant to clearly communicate a message between a specialist and a GP. The test and scan results primarily contain result values, but sometimes also additional comments. The general notes are about various patient interactions—telephone encounters, home visits, hospitalisation, etc.—but mostly they are about interaction with patients at a general practice. These kinds of notes are often divided into a part that describes what the patients said about their problems, and a part that records the GP’s train of thought during examination, which might variously include observations, conclusions, reflection on alternatives, and proposed further action. The two parts are commonly separated by a phrase or an acronym that roughly means “on examination”.

The general notes, as illustrated in Table 1, are written in a sub-language characterised by extreme brevity and telegraphic style of expression. The quality and presence of punctuation varies from completely missing to well placed commas and sentence markers. Spelling mistakes, abbreviated words or jargon, and frequent ungrammatical constructions are also present. Additionally, parts of the data have been redacted during the de-identification process, and replaced with tilde character strings. These characteristics make the notes challenging to fully comprehend for someone without medical training, and difficult to process by conventional NLP tools.

## Annotation design

When developing a new annotated corpus, one of the key decisions is whether to adopt an existing annotation scheme and guidelines or design new ones. Even though there is an established chunk annotated corpus, namely the CoNLL-2000 shared task corpus (Tjong Kim Sang and Buchholz 2000), there are no established guidelines for its chunk annotation scheme. Picking a particular annotation scheme for semantic entities also seems difficult as even though there are quite a few annotated resources, they are usually quite specific and dependent on the task they were designed to support. Perhaps the only exception to this is TimeML (ISO 2008), which was used in a number of cases as a basis for the scheme definitions and guidelines for temporal events.

Another important issue is the choice of annotators and their background. Roberts et al. (2009) present evidence that clinically trained annotators are better than linguists and computer scientists at annotating clinical records with semantic relations. However, there is no clear evidence that this is true for linguistic annotation such as chunking. On the other hand, Fan et al. (2013) uses linguist annotators for a syntactic annotation of malformed POS-tagged sentences. Ultimately the choice of annotators depends on the amount of effort and training that they would need to achieve comparable results. Our intuition was that chunking should be relatively easy to teach to medical students with basic understanding of grammar, while teaching linguists clinical vocabulary and some basic contextual knowledge seems like a difficult task. Therefore we chose to train fourth year medical students with substantial medical knowledge and sufficient experience with GP notes as annotators. However, achieving good results depended also on keeping the annotation as simple and clear as possible to minimise the required linguistic training. Therefore, we chose to develop our own scheme and guidelines for the Harvey corpus, based on a widely used annotation scheme.

The choice of suitable annotation tool was a more technical, but nonetheless important issue. We chose BRAT (Stenetorp et al. 2012), because of its clean and simple web-based interface, flexibility, and centralised data storage. It allowed us to give remote access to our annotators, while preventing them from copying the text they were annotating.^7^ It also logged the time stamp of all annotations, which allowed us to roughly track the time periods the annotators were working for.

This section describes in detail the design and refinement of the annotation scheme and guidelines. We developed them in a fashion similar to the CLEF corpus and guidelines (Roberts et al. 2009) which adheres to the principles of language resource annotation for information retrieval formulated by Boisen et al. (2000). First, we developed a draft version of the scheme and guidelines (see Sects. 4.1, 4.2), and then we refined them incrementally with the help and feedback of two medical students who became our first annotators (see Sect. 4.3). Finally, we trained another medical student to both be able to annotate text and adjudicate the annotations of the other two (see Sect. 4.5).

### Annotation scheme

The greatest challenge in the initial design of the annotation scheme was to find the appropriate balance between encoding enough information to support further research, and achieving clarity, simplicity, and conciseness in the guidelines. The annotation scheme had to capture as much syntactic structure as possible, while not “inventing” elements that were not there in order to create canonical structures. Adopting chunks as the main units of annotation was a logical solution as Abney (1991) defines them as “the parse trees that are left behind after we have unattached problematic elements”. In other words, chunking trades the levels of the parse tree closer to the root (the longer range relations) for better quality in the levels closer to the leaves (shorter range relations). But while chunking sacrifices information in standard grammatical text, it is appropriate for clinical notes, because there is less tree structure to be lost.

To our knowledge the only available comprehensive chunking guidelines were presented by Bharati et al. (2006), but they were designed for Indian languages and annotators with linguistic background. A more popular approach to chunking, is the pruning of full parse trees, as suggested by Abney (1991) and implemented by Tjong Kim Sang and Buchholz (2000) on a subset of the Penn TreeBank (Marcus et al. 1993) for the CoNLL-2000 chunking challenge. Given these circumstances we developed a new annotation scheme and a set of matching annotation guidelines acknowledging the telegraphic language style and many omitted words in the data. We also considered the background of the annotators, as they were expected to be native English speakers with limited understanding of linguistic theory and terminology such as parts of speech and syntax.

After the preliminary discussions we designed an initial annotation scheme and applied it to a few records to be able to discuss problems and possible improvements. The initial set of chunk types comprised of noun phrase chunks (NPs), adjectival phrase chunks (APs), main verbs (MVs), and prepositional phrase chunks (PPs). After a few iterations we made several alterations to the set of annotation types in order to make them clearer and simplify the task. Base noun phrase chunks were introduced because they allowed more flexible analysis than full noun phrases. Prepositional phrase chunks were excluded as many of them can be reliably recognised using pattern matching on top of NPs. The AP definition was altered to include only comparative expressions and predicative expressions such as *brown* and *better* in *My dog is brown* and *Patient’s tummy feels better*.

On another note, producing language resources such as the Harvey corpus requires significant amount of money, time, and labour, which prompted us to look for more annotation types that could be added to the scheme in order to make the annotation process more cost efficient. We introduced four types of semantic annotation as we thought they were likely to be useful in future research.

Quantity, frequency, and time of occurrence are important pieces of information not only for symptoms and diseases, but also for drug prescription and administration. They may contribute to symptom and disease recognition, and they are also useful for healthcare related research, such as studying drug side effects. *Quantitative expressions* (QE) cover all forms of the various quantities recorded in the data, such as *pulse* 90, 20 ml, etc. They should not be mistaken for identification numbers or any other non-quantities. The only quantities that are not annotated as QEs are units of time, e.g. 1 h. We define *temporal expressions* (TE) as words, phrases or clauses that contain information related to time. They can manifest as a reference to a specific moment in time (*in two days*), the duration of an event (*for two hours*), or an event’s frequency (*twice a day*). Even though using TimeML for clinical text was popularised with the last i2b2 challenge (Sun et al. 2013), we thought it would be overcomplicating the annotation scheme given that we did not intend to keep any connection between the records in the corpus. Location is also an important aspect of the information contained in the corpus. The location of the patient encounter (*home* vs. *clinic*) might be important, as well as the locus of a symptom (*joint pain*) or a disease (*lung cancer*). We introduced *locative expressions* (LE) to mark these two types of locations in the corpus. Finally, there are a number of expressions, such as *o/e*, that mark the border between patient narrative and the GP’s train of thought—we call them *on examination expressions* (OE). The ability to recognise such markers could provide contextual information. For example, speculative diagnoses before the marker are likely to be associated with the patient and after the marker with the GP. In this paper we refer to all four semantic annotation types described above as *expressions* or Named Entities (NE) even though very few of them contain any names.

We consider the two groups of annotations—syntactic chunks and semantic entities—to be two separate almost independent sets of annotations. Therefore, it is inevitable that annotation embedding will occur in some cases, and we had to provide a set of rules to govern this. Sequential taggers can assign only one label per token, therefore if there are parallel annotations there should be complex tags, which would greatly increase the size of the tagset as all combinations should be accounted for. In some cases such increase might be an acceptable trade-off, however, in our case the relatively small size of the corpus and its expected sparsity make a large tagset undesirable. To ensure that no embedding is done within the same tagset we introduced the following rule:

1. *Rule of structure simplicity:* no chunk annotation can be embedded in another chunk annotation, and no expression annotation can be embedded in another expression annotation.

Embedding annotations from different groups gives us the opportunity to make assumptions about the annotations, which may be helpful during the annotation process. We assume that all annotations should be representable as syntactic constituents and therefore if their boundaries overlap, one of them must contain the other. We introduced the following rule to reflect this assumption:

2. *Rule of compatibility:* embedding may occur only when the annotation borders coincide or when one of the annotations is inside the other (inclusive border indices)

**Fig. 2:**
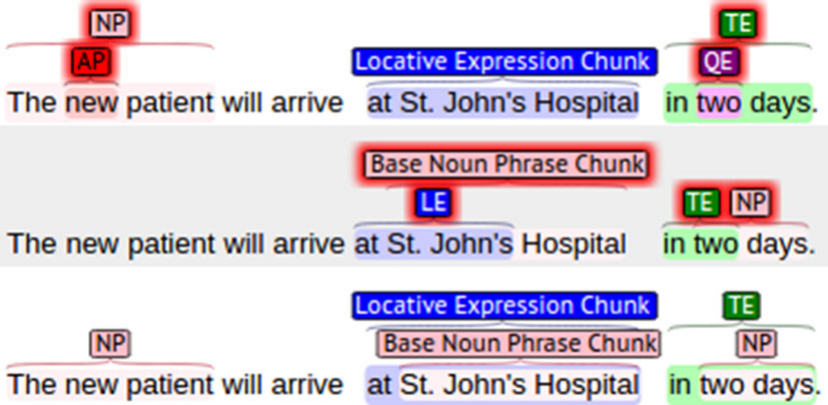
Examples illustrating correct (*line three*) and incorrect (*lines one* and *two*) use of embedded annotations

Figure 2 illustrates the correct and incorrect use of embedded annotations according to the rules defined above. The embedded annotations in the first sentence contradict the rule of simplicity: an AP is embedded in a NP, and a QE in a TE. The embedded annotations of the second sentence partially overlap each other without any of them fully containing another annotation. The annotations in the third sentence show the correct way of embedding complying with both rules.

### Annotation guidelines

We developed a document to describe the annotation types and explain how difficult cases should be treated, to ensure consistency. The goal was to design it as a training manual with enough examples so that it could be used as a reference during annotation too. It was meant to address the expected lack of linguistic knowledge of the annotators by giving a short introduction to English grammar.^8^

After an introduction to the project goals and expectations, the first part of the guidelines introduces the reader to basic grammar. It describes the concepts of phrases and parts of speech, concentrating on verbs, NPs, and APs in particular. The second part provides detailed definitions of the annotation types, along with examples and special cases that can be used as a quick reference manual during annotation. The last part of the guidelines helps increase the quality and consistency of the annotation by giving practical advice on some common issues and detailed instructions on how to handle particular situations—they urge the annotators to be confident in their opinion, while not annotating text they do not understand. The annotators are also encouraged to consider the likely content of redacted text in their analysis, and to annotate acronyms and abbreviations whenever they can be identified as chunks or expressions. Key issues such as punctuation, conjunctions, and embedding of annotation are also discussed in the final part of the guidelines, as well as basic usage of the BRAT platform.

**Fig. 3:**
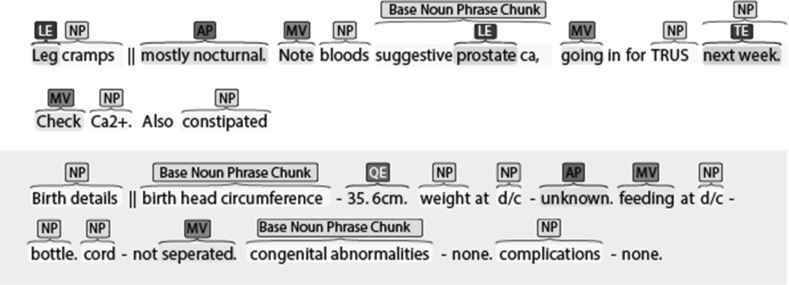
BRAT annotation showing labelled spans

BRAT allows the annotators to work with a web-based interface from a remote location (see part of the annotation window in Fig. 3), while preventing them from downloading any of the data. Finally, the guidelines describe the adjudication process and the role of the third annotator, which follows the example of Roberts et al. (2008) in restricting their duties to resolving annotation conflicts without adding or removing any information. The annotators are considered to agree when both of them have provided the same borders and tag for an annotation. In cases where only one annotation has been provided, it is considered to be correct as it is the only one available. The judge should intervene only in cases where candidate annotations overlap, using their own judgement to select the better annotation.

### Inter-annotator agreement

Inter-Annotator Agreement (IAA) was used as a quality metric and a source of feedback during the annotation development (see Sect. 4.5). The standard IAA evaluation metrics like Cohen’s *kappa* (Cohen 1960) and Krippendorf’s *alpha* (Krippendorff 2003) cannot be used, because both metrics rely on computing the probability of agreement or disagreement by chance, which are negligible due to the relatively unrestricted position and length of each annotation. Roberts et al. (2008) approached the issue by calculating the proportion of correct annotations with respect to the total number of annotations:

1 $$\begin{aligned} IAA = \frac{matches}{matches + {\textit{non-matches}}} \end{aligned}$$

Other studies (e.g. Alnazzawi et al. 2014) use an arguably more suitable approach adopting traditional information retrieval metrics as suggested by Hripcsak and Rothschild (2005). Similarly we adopted the MUC-7 test scoring rules (Chinchor 1998), which reflect the particular word span issues of our task (see Eqs. 2, 3). Five of the original six basic MUC-7 categories were used to calculate the scores. The non-committal category was not included in the calculations as it does not apply to our data.

2 $$\begin{aligned} Precision_{strict}= & {} \frac{Correct}{Correct + incorrect + partial + spurious} \end{aligned}$$

3 $$\begin{aligned} Recall_{strict}= & {} \frac{Correct}{correct + incorrect + partial + missing} \end{aligned}$$

**Fig. 4:**
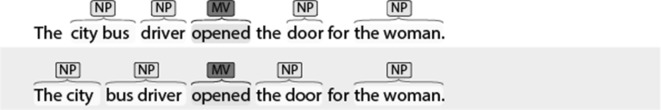
Two different annotations of the same text

We calculate *strict* and *relaxed* IAA, depending on the treatment of partial annotation matches. For example, *door* and *the door* in Fig. 4 are counted as incorrect NP annotations in strict mode (see Eqs. 2, 3), while the relaxed calculation counts them as correct (see Eqs. 4, 5) as long as one of the annotations completely contains the other. Overlapping annotations, such as *city bus* and *bus driver*, are considered mismatches by both calculation methods.

4 $$\begin{aligned} Precision_{relaxed}= & {} \frac{Correct + partial}{Correct + incorrect + partial + spurious} \end{aligned}$$

5 $$\begin{aligned} Recall_{relaxed}= & {} \frac{correct + partial}{correct + incorrect + partial + missing} \end{aligned}$$

We calculate the F_1_-score as the harmonic mean of precision and recall.

### Refinement

Inspired by the guideline development and refinement process described by Roberts et al. (2008), we set up a similar iterative process of annotation, evaluation, and refinement of the annotation scheme and guidelines. The plan was to send out small batches of 25–50 records to the annotators and analyse their results to improve the guidelines to a sufficient level. Our aim was to create a set of guidelines that would allow anyone to learn and produce a reasonable quality annotation with minimal in-person training. Such training was avoided initially in favour of independent self-training, because we believed that teaching by example might prevent the annotators from learning the appropriate linguistic generalisations.

The two domain specialists (annotators *A* and *B*) annotated fifty records remotely over the course of two weeks during the first annotation round. The agreement achieved only 0.35 F_1_-score, which is the lowest score measured throughout the experiments. An error analysis identified a few problems with the guidelines, including an ambiguity in the definitions of NPs and APs, which led to a many errors as they comprise a dominant part of the annotations. The two annotation types needed to be made more clearly distinguishable from one another. The basic grammar section had to be simplified, gerund definitions had to be added, and on-examination expressions needed to be specified as markers between text sections. The error analysis conclusions were also confirmed by feedback from the annotators. They suggested the examples in the guidelines should be improved and expanded. This prompted us to create an interactive tutorial using the BRAT platform showing definitions of all annotations with made-up examples, while testing the annotators against a solution key. During this refinement round very little was changed regarding the definitions of semantic entities, as the annotators did not feel confident in creating embedded annotations and annotated them sporadically.

The updated guidelines led to significantly better results in the second annotation batch. The agreement in all chunk categories and the on-examination expression improved, as well as overall agreement, which reached 0.43. However, there were considerably more instances of the other expression annotations, which decreased agreement in those specific categories even more.

Next we organised a workshop on the guidelines before the second refinement stage in order to gather more feedback from the annotators regarding unclear or insufficient information in the guidelines. We engaged the annotators in a series of discussions about each annotation type, stressing the relevant grammar points using non-medical examples and attempting to lead them to a correct understanding of the annotation through asking the right questions.

During the workshop it became obvious that the guidelines needed to explain the different roles of participles because the annotators experienced difficulty in distinguishing passive voice from adjectives, and continuous verb forms from gerunds. They also continued to avoid embedding different types of annotations, because the embedding rules were not clearly explained or illustrated by examples in the guidelines.

The third annotation batch had a steady overall improvement to 0.50 agreement in all categories except APs. The APs continued to be a confusing concept for the annotators, so APs were redefined to be as simple as possible, and an extensive range of examples was added. We also noted that even though certain aspects of the annotation improved and became more consistent, others worsened significantly in a way that could not be attributed to an ambiguity or lack of information in the guidelines. This made us look for other reasons why the annotators could be making errors. The BRAT platform log showed that the annotators worked on small 5–10 record subsets at a time, with breaks of at least a day between them. This confirmed a suspicion that the annotators were not fully concentrating when doing parts of the annotation, which often made them inconsistent. It became clear that it would be difficult to preemptively list all possible wrong interpretations of the guidelines and adjust the guidelines accordingly or warn the annotators about them. Thus even though the IAA results were improving, a change of training approach was required. We decided that the annotation scheme and guidelines had reached a stable level and our efforts should focus on setting up a productive environment for the annotation process.

### Annotator training

The observations we made during the first three annotation rounds suggested that the circumstances of the annotation process could be just as important as the training instructions. The annotators were always advised to work on as many records as possible in a single session, but during the first three batches they did not follow that advice, which resulted into many short annotation sessions with low consistency. Another observation, made by the annotators themselves, suggested that their understanding of the annotation deteriorates over time, for example during the 2-week gap between the second and third annotation batches. They also consistently found that the first few records in every session would take them more than the usual time and effort.

**Fig. 5:**
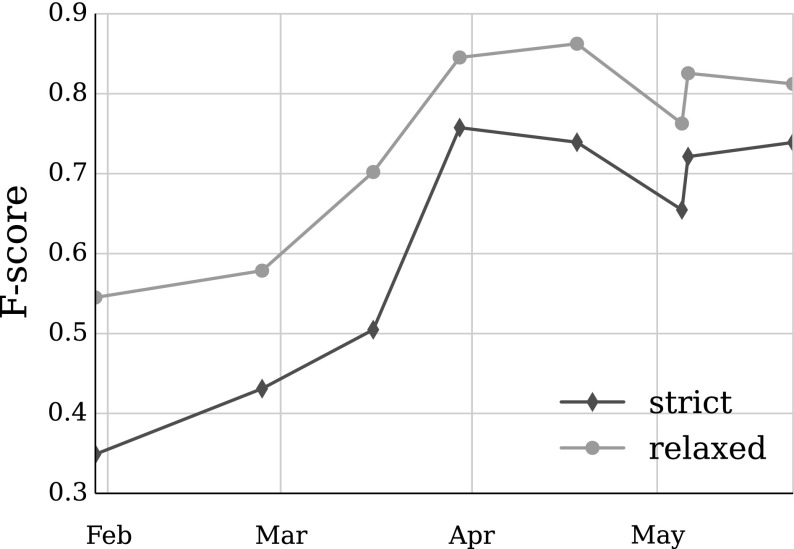
Inter-annotator agreement during the training period

We addressed these issues by setting up the annotation sessions in a university computer lab rather than at home, with one of the authors present to answer questions, restricted to the general interpretation of the guidelines. The new setup aimed to increase annotator concentration, while also introducing some training into the process by making them generalise their questions in order to receive answers. A week before the fourth annotation round, a short tutorial was organised to refresh their skills and to address some of the error patterns from the previous annotation rounds. The new annotation strategy resulted in a jump in the overall agreement to 0.76 F_1_-score, and a general increase in all separate categories, most notably in the chunks. Three out of the next four annotation sessions yielded similar results within 5 % points (see Fig. 5), which demonstrated that the annotators had achieved a sufficient level of consistency to start producing annotation for the corpus.

The training of the third annotator (referred to below as annotator *C*) started when annotators A and B had almost completed their training. The selected domain specialist was given a short introduction to the project and the guidelines before being assigned the first annotation batch. The annotation quality of the first batch appeared encouraging although it was hard to evaluate it using IAA as the annotation quality of the other annotators was low at the time. After the first batch, annotator *C* was given two more annotation rounds with feedback and took part in a workshop along with the other annotators at the end of the training phase.

**Table 3:** IAA between annotators *C* and *D* on their training annotation batches

	Strict			Relaxed		
	Precision	Recall	F_1_-score	Precision	Recall	F_1_-score
Chunks	0.65	0.64	0.65	0.82	0.80	0.81
Expressions	0.50	0.56	0.53	0.69	0.78	0.73
All	0.57	0.57	0.57	0.71	0.71	0.71

The results in *all* are calculated as micro-averages

**Fig. 6:**
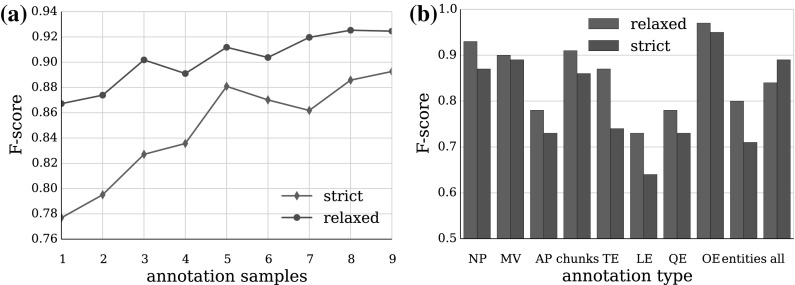
**a** IAA for the nine annotation batches of the corpus, in the order they were annotated; **b** IAA of the annotation types across the whole corpus

Unfortunately annotator *A* exited the project for personal reasons, and due to scheduling issues (the annotators were still students) was replaced by annotator *C* for the last three annotation batches of the corpus (see Fig. 6a). We trained a fourth annotator (annotator *D*) to both annotate and adjudicate as we did with *C*, although we used a slightly more hands-on approach with more detailed error feedback. Table 3 shows the IAA between *C* and *D* during their training period. The results are much higher than had been achieved by *A* and *B* in the development stage, but they are also lower than their results after the guidelines were completed (see Fig. 5).

**Table 4:** Pairwise IAA between all annotators

	Chunks						Expressions					
	AB	CD	AC	BC	AD	BD	AB	CD	AC	BC	AD	BD
Pr_*S*_	0.86	0.82	0.90	0.81	0.86	0.85	0.79	0.60	1.00	0.79	0.60	0.50
Re_*S*_	0.84	0.75	0.91	0.84	0.78	0.78	0.73	0.90	1.00	0.73	0.90	0.70
F_1__*S*_	0.85	0.78	0.90	0.82	0.82	0.82	0.76	0.72	1.00	0.76	0.72	0.58
Pr_*R*_	0.90	0.92	0.90	0.84	0.94	0.92	0.79	0.67	1.00	0.79	0.67	0.50
Re_*R*_	0.88	0.84	0.92	0.87	0.85	0.84	0.73	1.00	1.00	0.73	1.00	0.70
F_1__*R*_	0.89	0.88	0.91	0.86	0.90	0.88	0.76	0.80	1.00	0.76	0.80	.58

*S* and *R* subscripts stand for strict and relaxed agreement. *Columns* represent annotator pairs denoted with their letters

In Table 4 we present the pairwise IAA of all annotators on a small dataset. None of the annotators had seen the data before they annotated it. While still being the lowest, we note that the agreement between *C* and *D* has improved significantly after training. In fact, they score better when paired with the other annotators. The complete agreement between *A* and *C* on expressions may seem odd, but it can be explained by the fact that there are only 15 observations in the dataset. Such a low density is not unusual for the whole corpus as we will show in Sect. 5.3.

## The Harvey corpus

The Harvey corpus is a collection of linguistically annotated de-identified 750 primary care notes (around 17,656 words, 22,914 tokens) with three layers of linguistic annotation. The first layer contains POS tags automatically assigned by cTAKES (Savova et al. 2010). The second and the third layers consist of manually annotated syntactic chunks and semantic entities. The rest of this section provides a description of the data selection process (Sect. 5.1), a more detailed explanation of the text processing and data manipulation that produced a single coherent data structure (Sect. 5.2), and an analysis of the annotation statistics (Sect. 5.3).

### Data selection

The Harvey corpus data was randomly selected from three GPRD data samples obtained for PREP. These samples were compiled by selecting a number of patients with relevant diagnoses and retrieving all their records for the preceding year. Therefore, even though the Harvey source data has some diversity, it is not representative of the entire GPRD. Additionally, before the random selection, the data was filtered to remove all notes under five tokens, notes containing only test results or image attachments, and communication with specialists. The latter records were filtered out because the language of the letters is quite formal and detailed, which makes it completely different to of the notes.

### Data assembly

The Harvey corpus consists of a set of records, each one about a patient encounter. Each record consists of a Read code term, followed by a sequence of tokens. The records were tokenised in two stages—before and after the annotation. The first stage used simple, conservative rules to tokenise regular use of punctuation, while the second stage involved tokenisation rules that were more specific to the patterns in the text. The second stage also integrated information from the manual annotation layers to identify additional token borders. We evaluated different statistical POS tagging models on one hundred records manually annotated with the PennTreebank tagset by one of the authors. The model from the cTAKES NLP system (Savova et al. 2010) was selected for the tagging of the Harvey corpus as it achieved the best performance on this test set. Our choice was further supported by the observation that the model correctly tags some idiosyncratic medical abbreviations such as *c/o* (complains of). Finally, syntactic chunks and expressions were manually annotated as described in Sect. 4.

### Data analysis

**Fig. 7:**
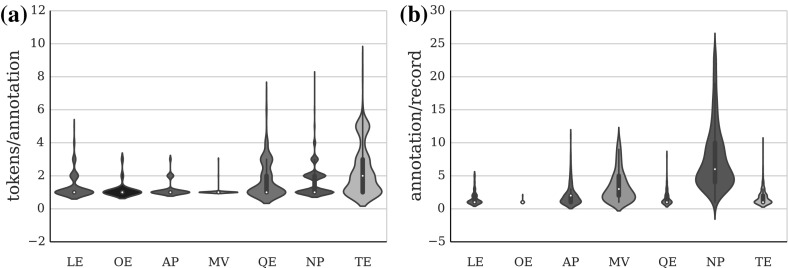
Arithmetic mean (*white dot*) and frequency distribution of **a** tokens per annotation, and **b** annotations per record, across all annotation types

Compared to popular clinical and biomedical corpora, the Harvey corpus is quite small (see Tables 2, 5), but still comparable to the those with linguistic annotation in the clinical domain (Pakhomov et al. 2004; Fan et al. 2011, 2013). On average, semantic entities are longer than chunks, which is to be expected from their definitions. QEs normally contain a quantity and a unit of measurement; TEs are very variable, ranging from short jargon expressions such as *2/7* (meaning two days), to full adjunct constructions like *a month before cancer diagnosis*; and OEs are dominated by the three character abbreviation *O/E*. LEs tend towards a single token average, because they typically occur as modifiers to a head noun in compound nouns such as *abdomen pain*, or abbreviated in one token—*ULQ* (upper left quadrant). Syntactic chunks tend to be short and frequent as a consequence of the telegraphic nature of the notes. The average number of tokens per chunk is below 1.5, which is indicative of a large proportion of single token chunk annotations. While this is to be expected from MVs and APs, the frequency and brevity of NPs certainly reflects the qualities of the data and its language (see Fig. 7a).

**Table 5:** Harvey Corpus statistics: annotation counts, average tokens per annotation, and average annotations per record

	NP	MV	AP	Chunks	TE	LE	QE	OE	NEs	All
Count	6304	2613	893	9810	605	481	321	73	1480	11,290

Another important aspect of the data is the gap between the frequency of NPs and the other annotation types. The figures in Table 5 suggest that only NPs and MVs are likely to occur more than 5 times in a single record.

The IAA shows a continuation of the positive trend from the training stage across the nine batches in which the corpus was divided for the annotation process (see Fig. 6a). The relatively large difference between the strict and relaxed agreement scores for most annotation types (5 % on average, see Fig. 6b) shows that a significant amount of the conflicting annotation could be overcome with minimal intervention during the adjudication process. This gives us further reason to believe in the good quality of the final corpus annotation. The agreement improvement varies from less than 1 % (OEs) to over 13 % (TEs) depending on the characteristics of the annotation types. Main verbs are much less prone to chunk boundary disagreement, because in most cases they are a single word. On the other hand, temporal expression boundaries could be difficult to identify with certainty in more complex cases such as periods of time (e.g. *more than six months*).

### Corpus availability

The data that the Harvey corpus was drawn from was obtained as part of the Patient Records Enhancement Programme under a license from the GPRD. Currently we are not permitted to share any of the data with anyone not covered by our license agreement. However, we are working towards a bid for the public or at least a less restrictive release of the data. Meanwhile we have made the annotation guidelines as well as the annotation (without the text) available on GitHub.^9^

## Extrinsic evaluation

The lack of an established quality metric for annotated corpora makes it difficult to compare and evaluate them. Therefore, corpora are often extrinsically evaluated through the impact they make on an application task. Following this methodology we set up experiments to evaluate the performance of two statistical models trained on Harvey corpus data: one for chunking, and one for named entity recognition. We also set up a comparison experiment using a randomly selected dataset (of size comparable to the Harvey corpus) extracted from the Penn TreeBank chunk data from CoNLL-2000. In all three experiments we used YamCha (Kudo and Matsumoto 2001, 2003), a state of the art SVM-based sequential tagger, to generate the models. The first two experiments aimed to establish if the corpus provides enough training data to achieve adequate results for the tasks of syntactic chunking and entity recognition. The third experiment aimed to compare the learning rates and the difference in performance between the Harvey data chunking model and one trained on edited text.

**Fig. 8:**
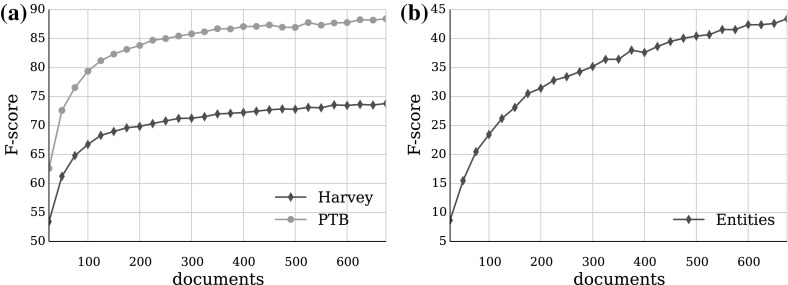
A 500-fold bootstrapping learning curves generated using YamCha: **a** chunking and **b** named entity recognition. Training samples range from 25 to 675 records with a step of 25; testing samples are always set to 75 records

Figure 8 shows the accuracy of the models estimated using bootstrapping (Efron and Tibshirani 1997) as the training data size increases. Instead of repeatedly analysing subsets of the data, as in cross-validation, bootstrapping repeatedly analyses sub-samples of it. Each sub-sample is a random sample with replacement from the full sample. The number of used sub-samples typically ranges from 50 to 2000 depending on the task and its goals. Each data point represents the mean F_1_-score of five hundred repeated evaluations using sub-samples of the data. As a result, the average standard error of the mean is relatively low: 0.14 % points for the chunks curve, and 0.30 for the semantic entities curve.

The shape of the Harvey chunking learning curve and the decreasing standard deviation suggest that the corpus contains consistent chunking annotation allowing a stable learning process. The increasing curve trend indicates that more training data should improve the performance, but it is difficult to predict to what extent. The difference with the edited text learning curve is large at the beginning, around 10 % points, and gradually increases to over fourteen and a half percentage points. This increasing difference suggests that it is unlikely that the Harvey curve will catch up given more data with the current training configuration of the model. However, Fig. 8 also shows that the current corpus size does not provide enough data to reach state of the art results even with regular text. If we assume that the trend in the learning curves remains, then the current chunking performance should increase from 0.74 F_1_-score (0.76 precision, 0.74 recall) to well over 0.80 if provided with the same amount of training data as the state of the art chunking models. There are also a number of factors that could easily improve the chunking performance. Our experiment did not try to adjust the training process in any way, but used the standard YamCha feature set for the CoNLL data; optimising features should help. Improving the quality of the POS tags of the Harvey corpus should also provide some improvement. We also expect that the proportion of unknown words encountered by the clinical data model is much higher than that of the Penn TreeBank model, which leaves more room for improvement through techniques tackling that issue.

The entity recognition model has a steeper learning curve, but a much lower performance at 0.43 F_1_-score with a significant gap between precision(0.69) and recall (0.32). These results are promising, because the distribution of the entity annotation is less balanced and much less frequent than that of the syntactic chunks, which is more uniform covering about 60 % of all tokens (see Table 5). A closer look at the results shows that locative expressions achieve only 25 % correctly tagged tokens, as opposed to over 90 % for on-examination expressions and 55 % for temporal and quantitative expressions. This can be explained by the very large vocabulary of the locative expressions, including body parts and regions expressed in both conversational and medical language style.

## Conclusion

We have produced a set of annotation guidelines and an annotated corpus of patient medical records consisting of physician-typed free-text notes and Read codes. In this article we have described the background, motivation, data source, annotation guidelines and procedures, and an evaluation of annotation quality.

Since the chunk annotations of most established language resources have been automatically generated rather than hand-annotated, the chunk annotation guidelines presented in this study are unique for the English language. They were planned as a self-sufficient tuition instrument for domain specialists, containing enough easily digestible linguistic knowledge to support the annotation process. The guideline development and the annotator training were set up as iterative processes, which returned in gradually improving agreement. We found that experience and longer annotation sessions improves IAA, while long periods of time between annotation sessions result in deterioration. After the training process was complete, inter-annotator agreement reached 0.86 F_1_-score for annotation of chunks, 0.71 for semantic entities, and 0.84 overall. The resulting parallel annotations of the corpus were combined by a third domain specialist resolving the conflicts with minimal intervention, producing the final version of the Harvey corpus, containing 750 records, 22,914 tokens, and 11,288 annotations. The corpus was extrinsically evaluated using two machine learning tasks. The experiments showed that performance increases with more training data and that the learning rate of the chunking classifier is comparable (but with a lower starting point) to a classifier using data from the CoNLL-2000 data set (see Fig. 8). In contrast, the named entity annotation is not enough for training an accurate classifier, as its F_1_-score reached only 0.43.

Despite these positive results, there are limitations to the Harvey corpus: relatively small size compared to other clinical text corpora, and lack of other important annotation layers such as parts of speech. Even though adding more data seems unlikely to increase chunking accuracy to levels seen with edited text, it is evident from the learning curves that it will continue improving it. Addressing other issues, such as POS tagging errors, should also decrease the chunking error rate, as its imperfect quality could have a harmful effect on the decisions made by the classifier. However, quantifying that effect requires a much more detailed analysis of the relation between the two. Such analysis should also optimise the features of the models for primary care data, as the configuration used in this study was the optimal YamCha configuration for the CoNLL-2000 data.

While the Harvey corpus is the first annotated language resource based on UK primary care text large enough to be used for developing machine learning tools, there are previous studies on US secondary care data with comparable goals. Both this study and that of Fan et al. (2013) are essentially aiming at adding syntactic information to difficult to process clinical text, but using different approaches and slightly different data. It will be difficult to compare results as there is free access only to the annotation, not the textual data of their study. However, the learning curve that we generated suggests that if more data is available the chunking accuracy should go well over 0.80, which is comparable to the performance of Fan et al.’s constituency parser. Even so, a fair evaluation would require an extrinsic measurement, such as impact on symptom identification, since chunking and constituency parsing are evaluated in very different ways.

In conclusion, the Harvey corpus provides a shallow parsing gold standard for primary care notes, which allows the development of accurate tools for syntactic chunking. The accompanying guidelines allow further annotation of more clinical data to be carried out in the same manner with similar annotation quality. The corpus and annotation guidelines will support future research in processing this kind of text and may serve as a foundation layer for annotating medication, symptoms, and diseases, which coincide with syntactic chunks. The potential benefits of such research should eventually minimise or even eliminate the need for manual processing and de-identification in typical information extraction tasks on UK clinical text. Development of such approaches will be essential to scaling up use of text, which has been shown to improve the quality of medical research

## Footnotes

1 The GPRD was renamed to Clinical Practice Research Datalink. http://www.cprd.com/.

2 General practitioners are the UK’s version of family physicians.

3 The corpus is named after William Harvey, the sixteenth century English physician and author of *De Motu Cordis*, also known as *On the Motion of the Heart and Blood*, the first mature account of the blood circulation process.

4 http://compbio.ucdenver.edu/ccp/corpora/obtaining.shtml.

5 The Oxford Medical Information System (OXMIS) was an earlier terminology system used in primary care computer systems from 1987. Practices switched over to the Read code system at different times in the 1990s (Shah et al. 2012).

6 The UK government has committed to migrating the primary care system to SNOMED CT by the end of 2016 and the whole NHS system by the end of 2020 (National Information Board 2014). Mapping tables between the standards can be found at https://isd.hscic.gov.uk/trud3/.

7 We implemented a small modification to the BRAT source that prevented the users from directly copying the text on their screens.

8 Some linguistic theory and explanations were simplified to make them more accessible to annotators without a linguistic background.

9 http://github.com/harvey-corpus.
